# 

**Published:** 1869-02-15

**Authors:** 


### CHICAGO MEDICAL JOURNAL

J. ADAMS ALLEN, M.D., LL.D., Editor. WALTER HAY, A.M., M.D., Associate Editor.

All letters for THE JOURNAL should be addressed to DR. J. ADAMS ALLEN, No. 71 Dearborn Street, Chicago, Ill.

TERMS—$3 PER ANNUM, IN ADVANCE.

$4 if not paid before 1st of July, in each year. Single Copies, 25 Cents.

